# Involvement of the Melanocortin-1 Receptor in Acute Pain and Pain of Inflammatory but Not Neuropathic Origin

**DOI:** 10.1371/journal.pone.0012498

**Published:** 2010-09-13

**Authors:** Ada Delaney, Margaret Keighren, Susan M. Fleetwood-Walker, Ian J. Jackson

**Affiliations:** 1 Medical Research Council Human Genetics Unit, Institute of Genetics and Molecular Medicine, University of Edinburgh, Western General Hospital, Edinburgh, United Kingdom; 2 Centre for Neuroregeneration, University of Edinburgh, Edinburgh, United Kingdom; University of Cincinnati, United States of America

## Abstract

**Background:**

Response to painful stimuli is susceptible to genetic variation. Numerous loci have been identified which contribute to this variation, one of which, *MC1R*, is better known as a gene involved in mammalian hair colour. MC1R is a G protein-coupled receptor expressed in melanocytes and elsewhere and mice lacking MC1R have yellow hair, whilst humans with variant MC1R protein have red hair. Previous work has found differences in acute pain perception, and response to analgesia in mice and humans with mutations or variants in *MC1R*.

**Methodology and Principal Findings:**

We have tested responses to noxious and non-noxious stimuli in mutant mice which lack MC1R, or which overexpress an endogenous antagonist of the receptor, as well as controls. We have also examined the response of these mice to inflammatory pain, assessing the hyperalgesia and allodynia associated with persistent inflammation, and their response to neuropathic pain. Finally we tested by a paired preference paradigm their aversion to oral administration of capsaicin, which activates the noxious heat receptor TRPV1. Female mice lacking MC1R showed increased tolerance to noxious heat and no alteration in their response to non-noxious mechanical stimuli. MC1R mutant females, and females overexpressing the endogenous MC1R antagonist, agouti signalling protein, had a reduced formalin-induced inflammatory pain response, and a delayed development of inflammation-induced hyperalgesia and allodynia. In addition they had a decreased aversion to capsaicin at moderate concentrations. Male mutant mice showed no difference from their respective controls. Mice of either sex did not show any effect of mutant genotype on neuropathic pain.

**Conclusions:**

We demonstrate a sex-specific role for MC1R in acute noxious thermal responses and pain of inflammatory origin.

## Introduction

The sensation of pain serves as a protective function, by alerting us to real or impending injury, triggering protective responses [1]. In a naïve animal, pain is initiated by direct thermal, mechanical or chemical activation of specific subsets of fine afferent C- and Aδ-fibre nociceptors. Individuals can differ in their perception of or response to pain. Within the human population, there are several rare, hereditary, sensory and autonomic neuropathies (HSANs) which are characterized by failure in development of or by degeneration of primary sensory and autonomic neurons [2]. Such conditions can lead to an impaired ability to perceive noxious stimuli and may result in permanent damage as normally painful injuries go unnoticed. There are also many non-pathological individual differences in nociception. It is well established that the sexes differ in their sensitivity to pain and in their responses to analgesia [3], [4], with females in general displaying greater sensitivity to pain than males [5], [6]. However this can be dependent upon genetic factors; for example in the Sprague Dawley rat strain, males are more sensitive than females to thermal nociception, while the opposite is seen in the Long Evans rat strain [7]. Analysis of morphine-induced analgesia further illustrates the complexity of sex in contributing to individual differences. In several strains of mice (AKR/J, C57BL/6J, SWR/J) males appear more sensitive to morphine, whilst in other strains there are no sex differences and in one strain studied (CBA/J) females are more sensitive than males [8].

Quantitative trait locus (QTL) mapping allows for the localisation of genes responsible for strain variability in nociception. A number of such loci have been reported among several different mouse strains, including *Nociq1 to 3* [9], [10], *Capsq1 to 4* [11], *Tpnr1* to *3* [9], [12], [13] and *Morph1 to 4* [14]. Two of the QTL reported, *Morph1* and *Morph4* co-localise with the strong candidate genes, *Oprk* and *Oprm*, which encode kappa (κ-) 1 and mu (µ-) 1 opioid receptors respectively[15]. A third opioid receptor gene, *Orpd1*, encoding the delta (δ-) opioid receptor, is also a strong candidate QTL, mediating strain dependent (C57BL6/J and DBA2/J) differences for thermal (hot-plate) nociception [12]. Another thermal nociception QTL, *Tpnr2*, localises to, and affects the expression of calcitonin gene-related peptide alpha (CGRPα), which has been shown to be involved in noxious heat sensitivity [13].

Analysis of κ-opioid-receptor agonist induced analgesia was found to be NMDA-dependent in male but not female mice [16]. A QTL underlying the strain difference between C57BL/6J and DBA2/J in kappa opioid analgesia was found on chromosome 8 in female but not male mice [17]. Mogil et al suggested that *Mc1r* may be a lead candidate gene underling this sex specific difference in analgesia [17]. Melanocortin-1 receptor (MC1R) is a G protein-coupled, seven transmembrane domain receptor that responds to small peptide hormones, derived from pro-opiomelanocortin (POMC) and belongs to a family of five receptors, MC1R to MC5R. The function of MC1R is best understood in the regulation of pigmentation through its expression in melanocytes. Loss of function mutations in mice and other mammals result in the synthesis exclusively of phaeomelanin, the yellow or red pigment of hair. Humans with reduced-function variants of MC1R have red hair. MC1R mRNA and protein are also expressed in the central nervous system, in the periaqueductal gray matter of the midbrain [18], an area known to be involved in pain modulation [19]. A translational approach was undertaken by Mogil and colleagues [17], providing evidence that MC1R plays a female-specific role in κ-opioid analgesia in *Mc1r^e^* mutant mice and correspondingly in humans with multiple MC1R variants. Further work has shown alteration in the basal sensitivity of these *Mc1r^e^* mutant mice, with an increased tolerance to thermal (heat) stimuli and in the tail clip and abdominal constriction tests [20] with no sex specific difference noted. Such alteration in basal sensitivity was replicated in humans, illustrated by a decreased sensitivity to electrical stimulus [20]. Additionally, the analgesic effects of clinically relevant µ-opioid analgesics were assessed in both mice and humans resulting in an increased analgesic response, again with no significant effect of sex [20]. Further characterisation of basal sensitivity in the human MC1R variant female population has shown a substantially higher sensitivity to cold pain, both perception and tolerance, and a slightly lower tolerance of heat pain [21]. In addition, red haired females showed decreased efficacy of subcutaneous lidocaine-induced analgesia, however male MC1R variants were not tested in this study [21].

Two of the melanocortin (MC) receptors, MC1R and MC4R, are unusual in that they have endogenous inverse agonists. All the MC receptors are activated by MSH (α, β, and γ forms; MC1R has the highest affinity for α-MSH) and adrenocorticotrophin (ACTH), which result in an increase in cAMP via coupling to G-alpha-S, leading to production of black or brown eumelanin. The endogenous inverse agonist, agouti signalling protein (ASP), normally expressed in the dermal papilla of the hair follicles, reduces MC1R signalling which leads to production of yellow/red phaeomelanin. MC4R is expressed in the paraventricular nucleus of the hypothalamus [22] and is involved in the regulation of appetite and feeding behaviour. MC4R also has an additional endogenous inverse agonist, agouti-related protein (AGRP). Gain of function mutations of the gene encoding ASP in the mouse can result in increased and widespread expression of the protein, leading to a yellow coat through action on MC1R, but also to obesity through ectopic inactivation of MC4R [23].

A number of additional physiological roles have been ascribed to MC1R, in particular in mediating the established anti-inflammatory activity of αMSH [24]–[26].

We have looked at the contribution of MC1R to naïve or basal nociceptive responses to noxious and non-noxious stimuli, and determined any sex differences in such responses, through the use of mutant (*Mc1r^e^*) mice. We also compared transgenic littermates in which the *Mc1r* deficiency has been rescued to identify if any alteration in behavioural responses could be rescued by the introduction of the human *MC1R* gene. We examined the contribution of *Mc1r* to pain states, of both inflammatory and neuropathic origin, again determining any sex differences in behavioural responses and whether introduction of human *MC1R* rescued the behavioural alterations observed in *Mc1r^e^* mice. In addition we have used ASP-over-expressing mice, (whereby MC1R and MC4R signalling is antagonised) to determine if blocking MC1R results in similar behavioural responses to a non-functioning MC1R. We finally looked at the contribution of MC1R to trigeminal nociception through oral aversion testing to capsaicin, which activates the noxious heat receptor, TRPV1 that is expressed in mouse tongue [27]. We determined any sex difference in MC1R's contribution to oral aversion and the outcome of rescuing MC1R function by introduction of human MC1R.

## Results

### Mutant and Transgenic Mice

In order to study the role of *Mc1r* in nociception in more detail we analysed the behavioural response to noxious and non-noxious stimuli of mice with a range of genotypes. Recessive yellow mice, *Mc1r^e^*, are homozygous for the *Mc1r^e^* allele and lack functional *Mc1r* by virtue of a single base deletion in the Mc1r gene which produces a frameshift at amino acid 183 and truncation of the receptor in the second extracellular loop between transmembrane domains 4 and 5. As a further comparison, and as a control for genetic background, we used littermates of the recessive yellow mice which have the mutant *Mc1r^e^* allele but also contain the human *MC1R* gene on a bacterial artifical chromosome [28]. These mice retain the normal regulation of human *MC1R* and have a wild type, agouti, coat colour. The mouse MC1R, in melanocytes at least, is able to signal independently of its cognate ligand a-MSH [29], [30], whereas the human MC1R requires activation by αMSH in both humans and in transgenic mice [28], [31], [32]. The endogenous inverse agonist of MC1R, ASP, blocks both ligand-dependent and basal, ligand-independent, signalling [28], [33]–[36]. ASP is normally expressed only in the skin where it modulates hair pigmentation. Mice with the dominant mutation *A^y-Jkn1^*, express ASP ubiquitously, and as a result have yellow hair, but are also obese due the action of ASP on the related receptor, MC4R. These mice were used to assess if antagonism of MC1R results in similar behavioural responses to mice with a non-functioning MC1R, by comparison to their non-mutant littermate controls (control non*-A^y-Jkn1^*).

### Naïve female *Mc1r^e^* mice have altered behavioural responses to noxious hot temperature

**Figure 1:**
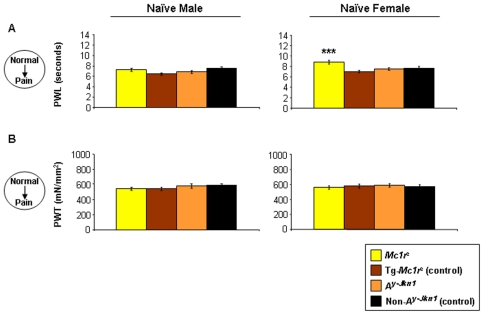
Behavioural responses to acute mechanical or noxious thermal stimuli in naïve animals. Adult male and female *Mc1r^e^* and their littermate controls Tg-*Mc1r^e^*, or *A^y-Jkn1^* and their littermate control non-*A^y-Jkn1^* mice were assessed for responses to acute noxious thermal or non-noxious mechanical stimuli. The paw withdrawal latency (PWL; n = 8–11 per genotype) [**A**], paw withdrawal threshold (PWT; n = 8–11 per genotype) [**B**] as measures of thermal hyperalgesia and mechanical allodynia respectively of both hind-paws (right and left hind-paw responses combined) in naïve animals are shown as mean responses ± SEM. Significant differences between *Mc1r^e^* and *A^y-Jkn1^* and their respective controls were only seen in female *Mc1r^e^* mice in [**A**] paw withdrawal latency (***P<0.01).

The behavioural responses of naïve male and female adult mice were assessed to noxious thermal and non-noxious mechanical stimuli. When we measured paw withdrawal latency in response to a noxious heat stimulus (Hargreaves' thermal stimulator) we found that female mice lacking *Mc1r* had an increased latency, indicating a higher tolerance to noxious heat, compared to female littermate mice with a normal mouse (data not shown) or human *MC1R* (Figure 1A). Male mice showed no variation by genotype in response to noxious heat stimuli. However we observed no difference in response to mechanical stimuli between genotypes or sex (Figure 1B). We also assessed the response of *A^y-Jkn1^* mice, which have a dominant agouti mutation and express ASP ubiquitously, and presumably signalling through MC1R is blocked wherever it is expressed. Surprisingly these mice differ from *Mc1r* mutant mice as they have a pain response indistinguishable from mice with a functional *Mc1r*.

### MC1R contributes to pain of inflammatory origin

**Figure 2:**
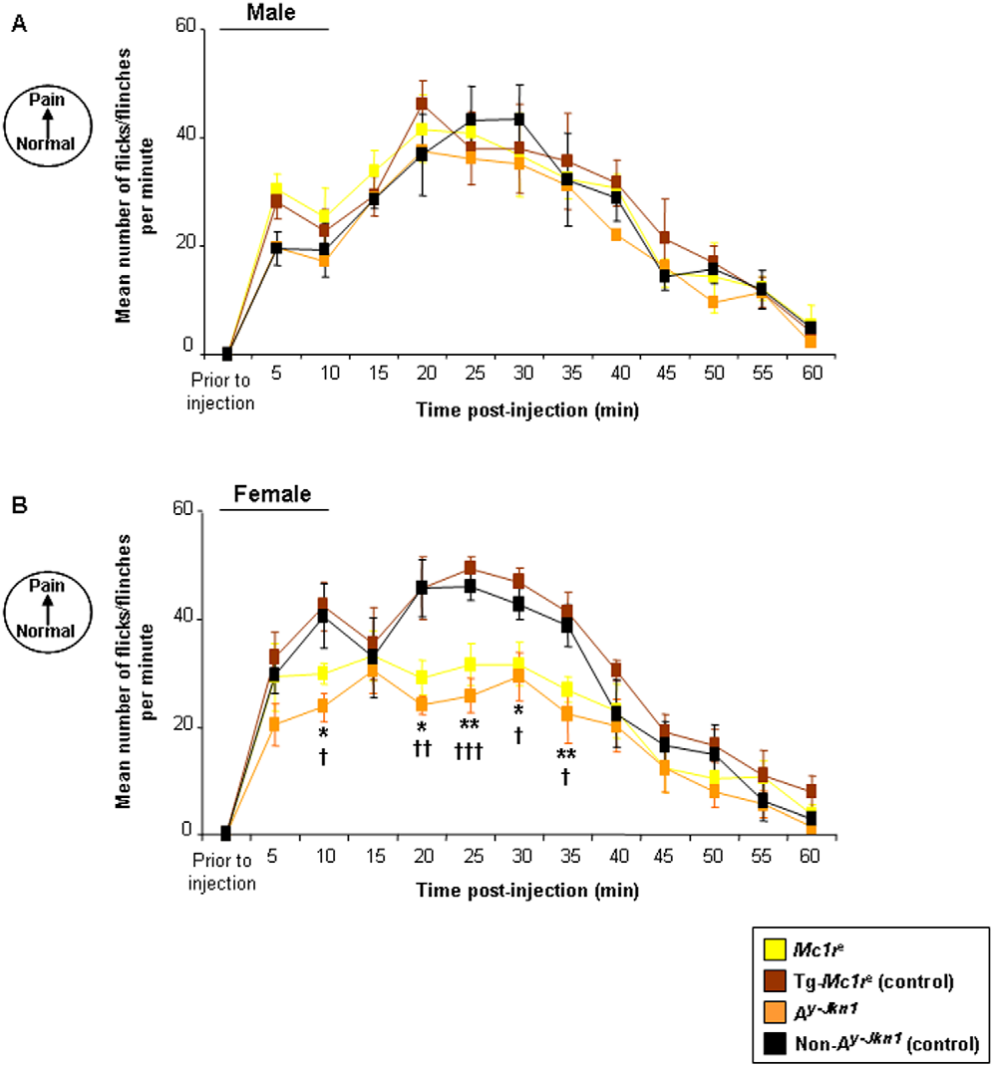
Effect of MC1R functionality on the Formalin Response. Data represents the number of paw flinch or flick responses per min ± SEM following the intraplantar injection of formalin in [**A**] male and [**B**] female adult *Mc1r^e^*, Tg-*Mc1r^e^*, *A^y-Jkn1^*and control non-*A^y-Jkn1^* mice (n = 5–9 per genotype). There was no significant difference in the formalin response occurring over time (0–60 min post-injection) for male *Mc1r^e^* or *A^y-Jkn1^* when compared to their control groups Tg-*Mc1r^e^* and control non-*A^y-Jkn1^* respectively. Significant differences for female *Mc1r^e^* or *A^y-Jkn1^* when compared to their respective control groups Tg-*Mc1r^e^* and control non-*A^y-Jkn1^* are shown *P<0.05; **P<0.01 (*Mc1r^e^* compared to Tg-*Mc1r^e^*) and †P<0.05; ††P<0.01; †††P<0.001 (*A^y-Jkn1^* compared to control non-*A^y-Jkn1^*).

We assessed the behavioural responses of these mice to inflammatory pain induced by injection of formalin into the plantar surface of one hind paw. The number of paw flicks or flinches was measured during both the first (5–15 min post-injection) and second (20–45 min post-injection) phase of the formalin response (Figure 2). Male mice of all genotypes did not differ in their response (Figure 2A). Female *Mc1r^e^* mice displayed a reduced response during both the first and second phase when compared to their littermate transgenic controls Tg-*Mc1r^e^* (Figure 2B). Interestingly, it appears that unlike noxious heat, the difference in formalin-induced inflammatory pain response can be modulated by inverse agonism by ASP. Female *A^y-Jkn1^* mice show a reduced first and second phase formalin response compared to control non- *A^y-Jkn1^* littermates suggesting that blocking of signalling through one or more melanocortin receptors reduces the formalin-induced inflammatory pain response.

**Figure 3:**
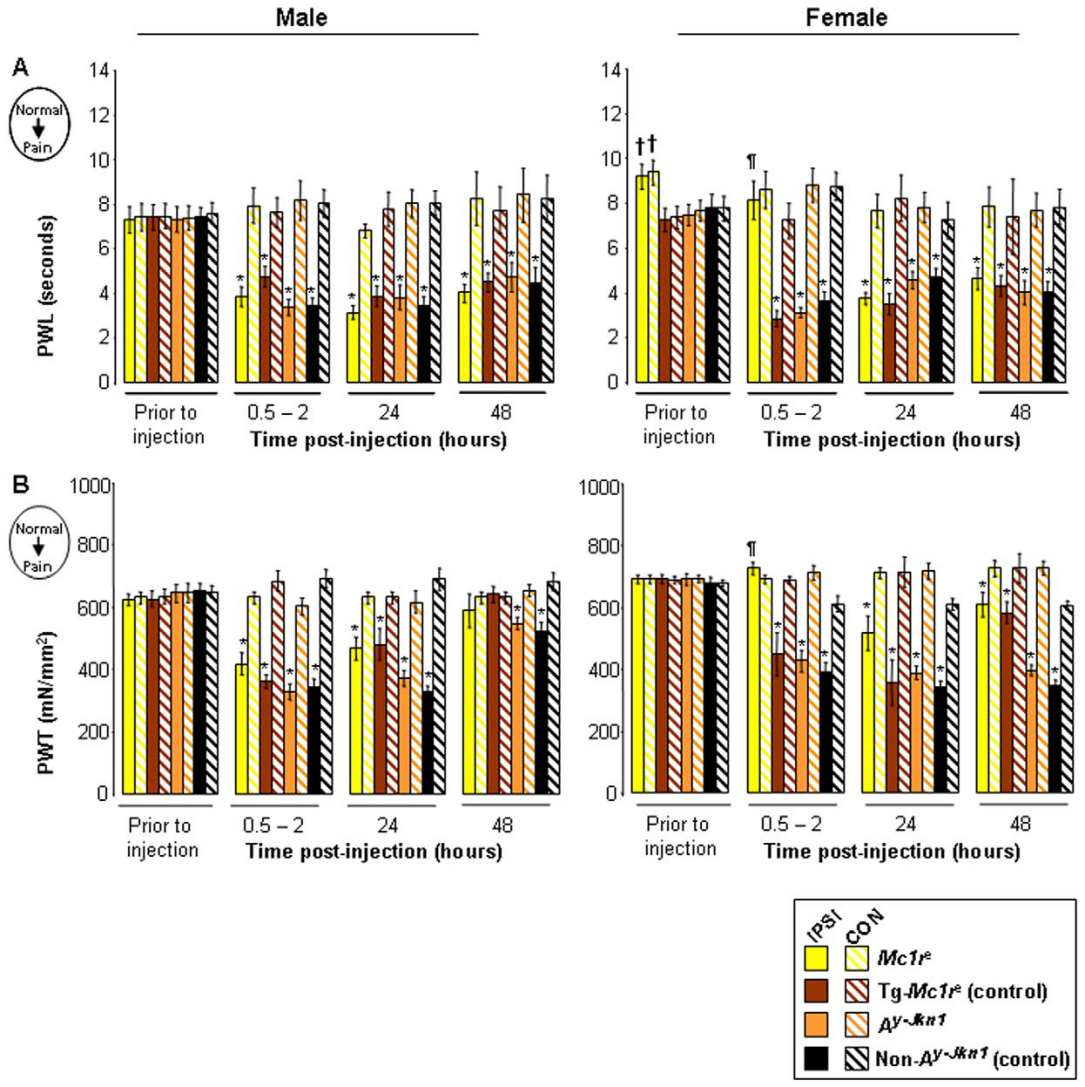
Effect of MC1R functionality on CFA-induced thermal hyperalgesia and mechanical allodynia. Data represents the paw withdrawal latency [**A**], paw withdrawal threshold [**B**] as measures of thermal hyperalgesia and mechanical allodynia prior to and post-injection of CFA (n = 4–5 per genotype), shown as mean responses ± SEM. Differences in behaviours prior to injection were observed in female *Mc1r^e^* mice only, when compared to their control littermates, Tg-*Mc1r^e^* in [**A**] paw withdrawal latency (††P<0.01). CFA-induced increased ipsilateral sensitivity (IPSI; injured hindlimb) compared to contralateral (CON; un-injured hindlimb) are shown (*P<0.05). Female *Mc1r^e^* mice were found to have a significantly higher ipsilateral thresholds when compared to their control littermates, Tg-*Mc1r^e^* ipsilateral thresholds in [**A**] paw withdrawal latency and [**B**] paw withdrawal threshold at 0.5–2 hours post-injection (¶P<0.05).

Injection of Complete Freund's Adjuvant (CFA) into the plantar surface of one hind paw, as a model of persistent inflammation, produces concomitant hyperalgesia (heightened pain response) and allodynia (lowered pain threshold). In male mice this response to inflammation can clearly be seen by increased sensitivity to thermal and mechanical stimuli; within a few hours of injection, the injured (ipsilateral) hind-paw's withdrawal from noxious heat has a reduced latency (hyperalgesia) (Figure 3A) and withdrawal from mechanical stimulation occurs at a much lower pressure (allodynia) (Figure 3B), when compared to the uninjured (contralateral) hind-paw. All genotypes of male mice had the same response.

Mutation of *Mc1r* delayed the development of thermal hyperalgesia and mechanical allodynia in females. Whilst other genotypes developed enhanced responses to noxious heat and mechanical stimuli within a few hours, *Mc1r^e^* females did not show development of inflammation-induced sensitisation until 24 hours post-injection. (Figure 3A, 3B).

### MC1R does not contribute to neuropathic pain

**Figure 4:**
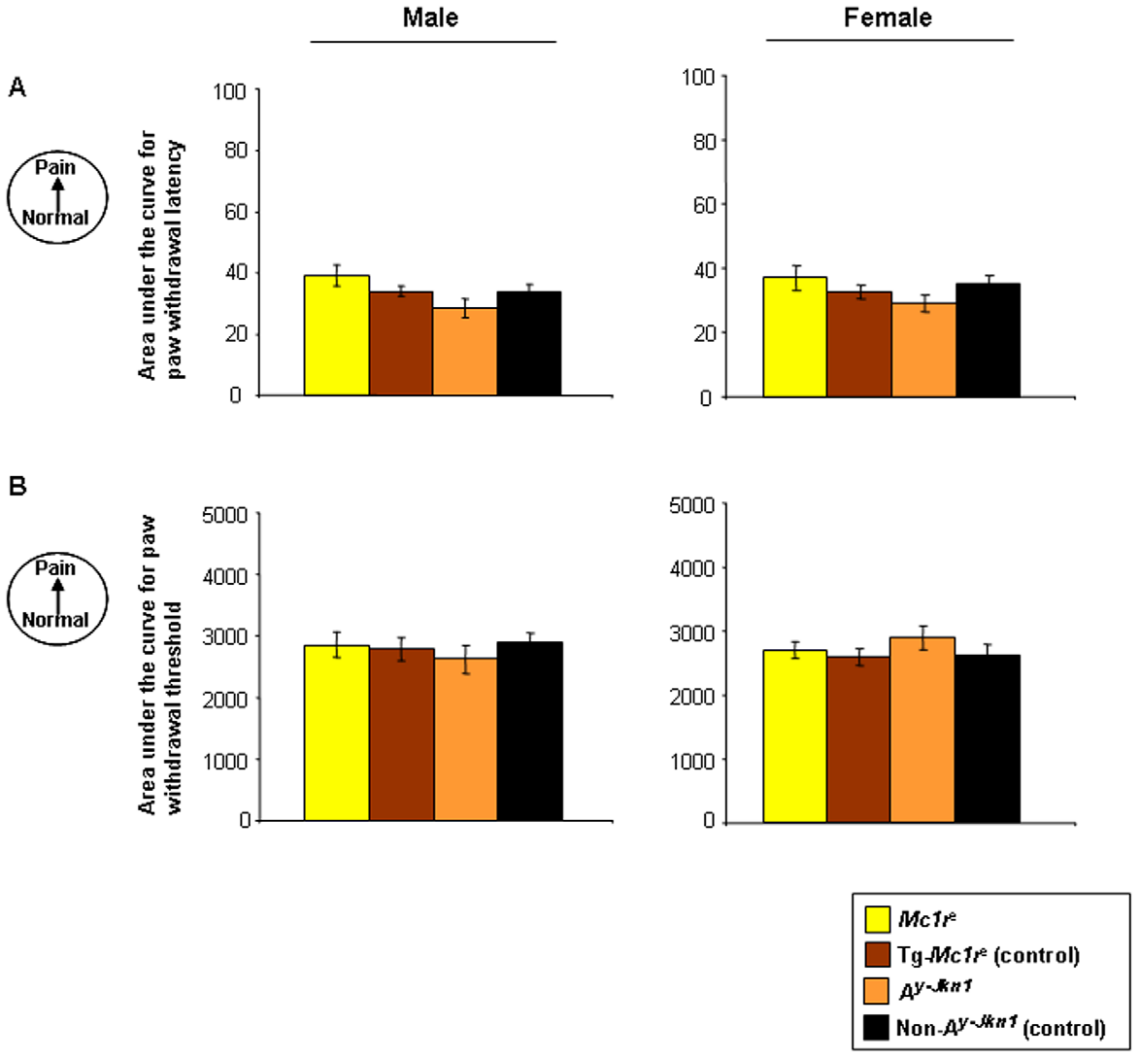
Behavioural analysis of CCI-induced thermal hyperalgesia and mechanical allodynia. Data represent the paw withdrawal latency [**A**], paw withdrawal threshold [**B**] as measures of thermal hyperalgesia and mechanical allodynia respectively in male and female *Mc1r^e^*, Tg-*Mc1r^e^*, *A^y-Jkn1^* and control non-*A^y-Jkn1^* adult mice, prior to- and post-surgery shown as the area under the curve (AUC; which measures the total change over the time course (baseline to post-surgery day 21) of CCI-induced thermal hyperalgesia and mechanical allodynia; n = 5–6 per genotype). AUC's were calculated from raw data by the standard trapezoidal rule. No significant differences in either male or female mice were observed.

In order to test the effect of MC1R on neuropathic pain we used a chronic constriction injury (CCI) by loosely ligating the sciatic nerve of mice [37]. Paw withdrawal latency in response to noxious heat and the paw withdrawal threshold in response to mechanical stimulus were measured on the injured hindlimb (ipsilateral) and compared to the un-injured (contralateral) hindlimb as a control. In all genotypes and both sexes there was a striking development of thermal hyperalgesia and mechanical allodynia following surgery, which resolved over the following 40 days. There were no differences between genotypes in either sex, indicating that in contrast to inflammatory pain, Mc1r does not appear to play a role in neuropathic pain (Figure 4).

### MC1R affects trigeminal nociception of chemical stimuli

**Figure 5:**
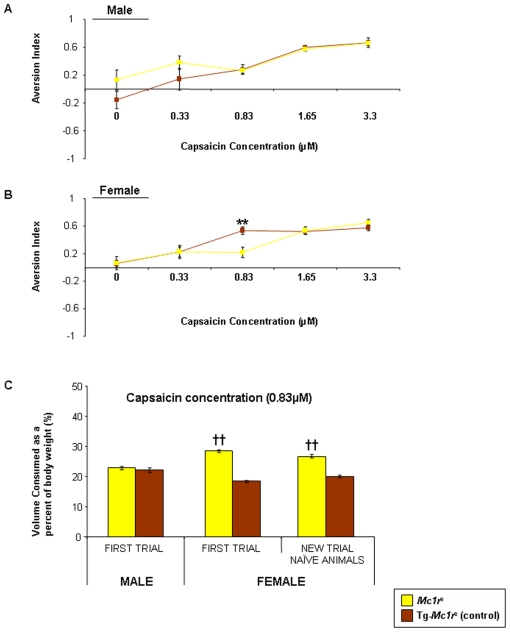
Concentration-dependent aversion to capsaicin consumption. Concentration related aversion to capsaicin in adult [**A**] male and [**B**] female *Mc1r^e^* and their control littermates Tg-*Mc1r^e^* (n = 6–7 per genotype). Data is expressed as an aversion index ranging from −1 to 1, which measures the degree to which the solution was avoided during the 10 day exposure at each capsaicin concentration tested ± SEM and was calculated by subtracting capsaicin consumed from water consumed, differences in *Mc1r^e^* consumption of capsaicin are shown (**P<0.01) compared to control Tg-*Mc1r^e^* group. [**C**] Shows the concentration threshold for capsaicin (at 0.83 µM) in the first trial of male and female mice, followed by a repeat trial in naïve animals (not previously assessed for oral aversion) data is expressed as the volume capsaicin containing solution consumed as a percentage of body weight ± SEM during the 10 day exposure, differences in *Mc1r^e^* consumption of capsaicin at 0.83 µM are shown (††P<0.01) compared to control Tg-*Mc1r^e^* group.

The hindlimb withdrawal responses to noxious and non-noxious stimuli, is mediated through the lumbar dorsal root ganglia and spinal cord. Responses in the head and face region are mediated through the trigeminal ganglia. The receptor responsible for detection of noxious heat (TRPV1) responds to capsaicin (the active compound in chilli). We asked if MC1R played a role in response to this receptor through the trigeminal ganglia using an oral aversion test. We offered mice lacking *Mc1r* and transgenic, human *MC1R*-positive littermate controls, a choice of water or highly dilute capsaicin, and assessed their consumption over a period of 10 days. The capsaicin dose was increased after each period. As the dose increased, relative consumption of the capsaicin solution decreased, indicating that the mice find it unpalatable. At higher concentrations (1.65–3.3 µM) all mice were equally averse but we noted a threshold concentration of 0.83 µM at which there was a genotype-specific difference (Figure 5A, 5B). We tested a second cohort of naïve mice at this concentration and replicated this difference in consumption. As might be predicted from their higher tolerance of noxious heat, female *Mc1r^e^* mice consumed a much higher proportion of capsaicin than their transgenic, control littermates Tg-*Mc1r^e^* (Figure 5B, 5C). Male mice of either genotype consumed an equal proportion of the capsaicin solution (Figure 5A, 5C).

The results of all the nociception assays performed are summarised in Table 1.

**Table 1:** Summary of Results.

	MALE		FEMALE	
	*Mc1r^e^* compared to *Tg-Mc1r^e^*	A^y-Jkn1^ compared to control non-A^y-Jkn1^	*Mc1r^e^* compared to *Tg-Mc1r^e^*	A^y-Jkn1^ compared to control non-A^y-Jkn1^
Acute test (naïve animals)				
Noxious heat	no difference	no difference	increased threshhold	no difference
Mechanical	no difference	no difference	no difference	no difference
Formalin response (tonic inflammation)				
Response	no difference	no difference	decreased response	decreased response
CFA (model of persistent inflammation)				
Noxious heat	no difference	no difference	delayed sensitivity	no difference
Mechanical	no difference	no difference	delayed sensitivity	no difference
CCI (model of peripheral neuropathy)				
Noxious heat	no difference	no difference	no difference	no difference
Mechanical	no difference	no difference	no difference	no difference
Capsaicin oral aversion (activation of noxious heat receptor)				
Aversion	no difference	not tested	reduced aversion	not tested

Results are summarized for adult male and female mice comparing; ***Mc1r^e^*** *,* recessive yellow mice with their littermate controls **Tg-** ***Mc1r^e^*** and ***A^y-Jkn1^***, agouti signalling protein over-expressing mice with their littermate controls, **control non-** ***A^y-Jkn1^***. All mice were tested for behavioural responses to acute (noxious thermal or mechanical stimuli) tests, formalin response, CFA and CCI-induced thermal hyperalgesia and mechanical allodynia, oral aversion was tested in *Mc1r^e^* and Tg-*Mc1r^e^* mice only.

## Discussion

Previous work has demonstrated a role for MC1R in basal pain perception and in response to analgesia in both mice and humans. Most studies have found that only females demonstrated an effect of mutant MC1R. We have shown here that, in our assessments of acute pain perception, female, but not male, *Mc1r* mutant mice (*Mc1r^e^*) have an altered response to noxious thermal stimuli, displaying an increased tolerance of noxious heat stimuli. We have extended the analysis to examine formalin-induced inflammatory pain and the heightened pain perception to thermal and mechanical stimuli as a result of CFA-induced inflammation. The formalin model of inflammation is a well characterised test [38], which has been shown to elicit a biphasic nociceptive response, an acute/first phase (lasting for 5–10 minutes following injection), and a tonic/second phase (lasting from 20–40 minutes until the nocifensive response diminishes to baseline). The acute phase of the formalin response is thought to be produced by direct excitation of nociceptors at the site of injury, while the second phase involves both ongoing peripheral activity and central sensitisation in the spinal cord [39]. While licking/biting behaviours are a more dependent measure of the formalin response we have looked at the number of flinch/flick (lifting) behaviours, which may account for the reduced interphase responses observed [40]. Here female *Mc1r^e^* mutant mice have a decreased nocifensive response during both phases of formalin-induced inflammation that is also observed in female *A^y-Jkn1^*, indicating that antagonism of melanocortin receptors in addition to a non-functional MC1R results in reducing the formalin response. The role of MC4R in reducing the formalin response cannot be ruled out in the *A^y-Jkn1^* mice; indeed the second phase of the formalin response has been shown to be attenuated by antagonists that preferentially act on MC4R [41]. In addition, inflammation-induced hyperalgesia and allodynia following intra-plantar injection of CFA is delayed by 24 hours, such that the injured hindlimb of *Mc1r^e^* females does not initially differ in sensitivity to the un-injured hindlimb. However CFA-induced hyperalgesia and mechanical allodynia is apparent at 24 and 48 hours, with the same degree of behavioural sensitisation observed as with Tg-*Mc1r^e^*, *A^y-Jkn1^* and control non*-A^y-Jkn1^*. Interestingly although there is no behavioural effect of mutant MC1R on naïve mechanical sensitivity, the onset of mechanical allodynia in this model of persistent inflammation is delayed. The delay in thermal hyperalgesia is reflective of the basal sensitivity, where an increased tolerance to noxious heat is found. It appears that inflammation-induced hyperalgesia corresponds to basal sensitivity of the *Mc1r^e^* mice, perhaps indicating a potential role for MC1R involvement in TRP channel activation or signalling that may be continued post-injury.

Previous work has shown that antagonism of MC1R alone results in behavioural responses similar to *Mc1r^e^* mice [17]. However whether the absence of effect observed in the *A^y-Jkn1^* mice is a result of dual antagonism of the MC1R and MC4R would need to be further investigated (for example through use of MC4R antagonists in the *Mc1r^e^* mice). Furthermore, the possibility that other melanocortin receptors might compensate when both MC1R and MC4R are blocked cannot be ruled out. It is also worth noting that the *A^y-Jkn1^* mice are obese as a result of inhibiting the MC4R, and the role that this has to play in terms of behavioural reflex assessment of the hindlimb needs further clarification. It is interesting that in these mice the basal sensitivity (assessed through use of quantitative sensory testing, which relies on application of thermal or mechanical stimulus to the plantar surface of the hindpaw), shows no change. The formalin response results in spontaneous pain-related (ie nocifensive) behaviours and is therefore independent of plantar stimulus application. When such a spontaneous response is assessed the behavioural response seen in the *Mc1r^e^* mice is replicated in the *A^y-Jkn1^* mice, with no alteration observed in the control non*-A^y-Jkn1^* littermates. MC1R has been extensively reported to have a role in mediating anti-inflammatory activity of αMSH [24]–[26], indeed αMSH has been reported to reduce the formalin response [42]. The role of this receptor in behavioural responses here appears to differ from these findings, with inactivity of the MC1R not resulting in a normal or pro-inflammatory response but rather a reduced inflammatory nocifensive response.

Conversely we report a lack of effect of mutant MC1R on chronic pain of neuropathic origin. MC4R, which is expressed both peripherally in the dorsal root ganglia [43] and centrally in the spinal cord and brain [44], has been reported as playing a role in neuropathic pain through use of antagonists (that are more selective for MC4R over MC3R) following peripheral nerve-injury, resulting in reversal of injury-induced behavioural sensitisation [45]–[47]. We find here that neither antagonism of the MC1R nor a non-functional MC1R influences nerve-injury induced sensitisation, with all groups having a similar time course of development of nerve-injury induced sensitivity. Additionally no alteration as a result of strain or sex was noted. The expression of MC4R has been shown to increase in the spinal cord after peripheral nerve-injury [47]. Following axotomy, no alteration in MC1R mRNA expression is seen in dorsal root ganglia and decreases in both MC2R and MC3R, but MC4R mRNA increases following axotomy [48], perhaps suggesting a greater role for MC4R than MC1R in nerve injury. However, we observed no alteration or reduced development of nerve-injury induced behavioural sensitisation in *A^y-Jkn1^* mice, where MC4R function is also blocked by the inverse agonist ASP. Perhaps the balance between agonist and endogenous inverse agonist activities is crucial to determining how the melanocortin system interacts with or regulates nociceptor function.

To further explore the potential involvement of MC1R with TRP channels in addition to trigeminal nociception, we used a two-bottle paired preference paradigm oral aversion test [49]. This test was used to test aversion to a chemical compound known to activate the TRP channel; TRPV1, which is selectively activated by noxious heat (>43°C) or capsaicin [50]. We have shown an increased tolerance to noxious heat in female *Mc1r^e^* mice. Using these mice and their control littermates Tg-*Mc1r^e^* we investigated whether aversion to capsaicin would also be altered by mutant MC1R. We found increased tolerance to heat can be extended to increased tolerance to capsaicin in female *Mc1r^e^* mice only. We repeated this assessment in an independent set of naïve male and female *Mc1r^e^* and Tg-*Mc1r^e^* mice and found this increased tolerance was repeated. This suggests the role for MC1R in TRP activation or signalling extends to chemical activation as well as thermal.

In summary we provide further evidence for the role for MC1R not only in acute thermal sensitivity but also in pain of inflammatory origin that is female specific. The existing data for the role of MC1R in the human population appears to differ in outcome for tolerance of thermal stimuli [20], [21]. Our study provides additional evidence that mutation of MC1R contributes to an increased tolerance to thermal stimuli, similar to data presented by Mogil and colleagues [20]. While an altered response to cold and heat has been examined in humans [21], to test the conservation of this reported behavioural response to humans, it would be interesting to examine basal sensitivity to a range of temperatures and capsaicin sensitisation in both male and female MC1R human variants. We suggest an interaction between MC1R and TRP channel activation or signaling may occur but whether this may occur through direct or indirect activation needs to be examined. We highlight further the importance of MC1R in female pain modulation.

## Materials and Methods

### Animals, surgery and experimental groups

All experiments were carried out in accordance with the UK Animals (Scientific Procedures) Act, 1986, and approved by the University of Edinburgh Ethical Review Committee and performed under UK Home Office project and personal licences. Adult male and female mice of four genotypes were used in this study, summarized in Table 2. Recessive yellow mice, *Mc1r^e^*, lack a functional MC1R. To control for genetic background, Tg-*Mc1r^e^*, are littermates of the *Mc1r^e^* mice, which contain human *MC1R* gene on a bacterial artificial chromosome [28], leading to rescue of the non-functioning MC1R such that the yellow mutant pigmentation becomes a normal agouti coat colour as previously described [28]. Mice with a dominant mutation of agouti, *A^y-Jkn1^*, in which ASP (the endogenous inverse agonist of MC1R) is ubiquitously expressed and results in a yellow coat colour were used to assess antagonism of MC1R (these mice are also obese due to ASP's action on MC4R). Non-mutant littermates of *A^y-Jkn1^,* control non*-A^y-Jkn1^*, have a black coat colour due to the nonagouti allele, do not express ASP and were used as controls for *A^y-Jkn1^*. All animals were housed in groups (except in the oral aversion tests) at a temperature of 21°C ±1°C with a 12/12 hour light/dark cycle and had *ad libitum* access to food and water.

**Table 2:** Experimental Groups.

Strain Name	Hair Colour	Genetic Composition
*Mc1r^e^*	yellow	lacks functional MC1R
Tg-*Mc1r^e^*	agouti	nonfunctional MC1R rescued with human MC1R transgene
*A^y-Jkn1^*	yellow	normal MC1R, antagonized by ASP overexpression
control *A^y-Jkn1^*	black	normal MC1R but no ASP expression

Adult male and female mice used in this study were; ***Mc1r^e^*** *,* recessive yellow mice, which are null mutants with a non-functional MC1R resulting in a yellow coat colour and their littermate controls **Tg-** ***Mc1r^e^***, which are recessive yellow mice whose non-functioning MC1R is rescued with human MC1R and have an agouti coat colour. ***A^y-Jkn1^*** are agouti signalling protein (ASP) over-expressing mice with a functioning mouse MC1R resulting in a yellow coat colour and their littermate controls, **control non-** ***A^y-Jkn1^*** **,** are mice that do not over-express ASP and have a functioning mouse MC1R and a black coat colour.

**Formalin** was used to induce an experimental model of inflammatory pain [38] Under aseptic conditions formalin (10 µl of a 1.5% solution in saline; Sigma) was injected into the plantar surface of one hind paw under halothane/O_2_ anesthesia. Responses were characterised as the number of flinches/flicking (lifting behaviour) over 1 min in the injected hindpaw and recorded at 5 min intervals until 60 min post injection. Results are expressed as the mean number of paw flinches or flicks per minute ± SEM. Sample sizes were 5–9 per genotype.

**Complete Freund's Adjuvant** was used to induce an experimental model of persistent inflammatory pain [51]. Complete Freund's adjuvant (CFA 1 mg/ml; Sigma) was injected (1 µl/gram body weight) under aseptic conditions into the plantar surface of one hind paw under halothane/O_2_ anesthesia. Sample sizes were 4–5 per genotype.

**Peripheral Nerve Injury** was induced under aseptic conditions using a unilateral chronic constriction injury (CCI) [37], an experimental model of neuropathic pain, whereby the sciatic nerve was exposed and loosely ligated with 3 chromic catgut ligatures (5/0, Ethicon) placed about 1 mm apart under halothane/O_2_ anesthesia. Sample sizes were 5–6 per genotype.

### Behavioural Testing

Quantitative sensory reflex testing of mechanical allodynia and thermal hyperalgesia was performed on naïve, CCI and CFA injured animals (n = 8–11 per genotype), prior to surgery or injection to obtain baseline values and post-surgery/injection. Mechanical allodynia was assessed as paw withdrawal threshold (PWT), using calibrated von Frey filaments (Stoelting) applied to the plantar surface of the hindpaw, to determine the indentation pressure (mN/mm^2^) required to elicit a response (defined as the pressure required to elicit a paw withdrawal to at least 50% of applications, i.e. 4 out of 8 applications). Thermal hyperalgesia was measured using the Hargreaves' thermal stimulator (Ugo Basile) that applies a quantified noxious radiant heat to the plantar surface of the hindpaw, to monitor the paw withdrawal latency (PWL) in seconds [52]. Behavioural responses were recorded both ipsilateral and contralateral to injury pre- and post-surgery/injection, with responses obtained at each time point presented as the mean ± SEM.

### Oral Aversion Test

To evaluate the contribution of MC1R to trigeminal nociception, we used a paired preference paradigm aversive drinking test in *Mc1r^e^* and Tg-*Mc1r^e^* male and female adult mice to assess oral aversion to capsaicin. Capsaicin stock solutions were prepared as a 3.3 mM stock solution of capsaicin (Sigma) by dissolving in 30% ethanol and serial dilutions of the stock produced working concentrations of 0.33 µM, 0.83 µM, 1.65 µM and 3.3 µM. The paired preference paradigm set up was used to assess capsaicin induced oral aversion as described previously [49]. Briefly, each trial involved two water bottles, one containing capsaicin at the given concentration and one bottle containing water with ethanol at appropriate concentrations (to match the concentration found in the test solution, with the maximum concentration of ethanol (0.16%) not equalling that of either the maximum capsaicin solution to prevent the possibility of intoxication). All mice were housed one per cage on a 12/12 hour light/dark cycle, with *ad libitum* access to food and both bottles. To measure volumes consumed bottles were weighed (during the same 2 hour time point of 10am–12pm) on alternate days over a 10 day test. In order to prevent a position preference, the position of the bottles were switched at each weighing, so each solution was positioned on the right or left position for an equal number of days. On completion of the 10 day test all mice had free access to two new water bottles (containing water) for a minimum of 4 days before the next higher concentration of capsaicin solution was tested. A control cage was set up as outlined without any animal, to control for any spillage from both bottles on movement of cages and to control for possible evaporation of solutions. Raw consumption weights for each bottle were expressed as a percentage of total volume consumed for each group at each concentration. An aversion index was calculated as per Simons et al. [49], by subtracting the percentage of capsaicin consumed from the percentage of water consumed, resulting in an aversion index range of −1 to +1. Using this aversion index, −1 indicates capsaicin is wholly preferred to water, 0 indicates equal preference and +1 indicates water is wholly preferred. Data is presented as the aversion index as a function of capsaicin concentration. When significance in oral aversion to a particular concentration of capsaicin was found data is also presented as the raw consumption weights as a percentage of body weight ± SEM for each group at that concentration. Sample sizes were 6–7 per genotype.

### Analysis

Statistical analysis of data (presented as means ± SEM) was carried out as follows, with significance set at p<0.05. Paw withdrawal latencies were analysed by one way repeated-measures ANOVA with Bonferroni post-hoc test, paw withdrawal threshold were analysed by Friedman repeated-measures ANOVA on ranks with Bonferroni post-hoc test. Injury-induced ipsilateral differences (compared to contralateral values) were measured by student's t-test for paw withdrawal latencies and by Mann-Whitney rank sum test for paw withdrawal thresholds. Formalin response (genotype × response) and oral aversion (genotype × concentration) were assessed by two way ANOVA with Bonferroni post-hoc test. Oral aversion results presented at a single concentration were analysed by one way ANOVA with Bonferroni post-hoc test at that concentration. Areas under the curve (AUC) for CCI-induced mechanical allodynia and thermal hyperalgesia were calculated from raw data converted to AUC (to post-surgery day 21) using the standard trapezoidal rule, differences were determined by one-way ANOVA with Tukey's post-hoc test.
